# Recent Developments of Carbonic Anhydrase Inhibitors as Potential Drugs

**DOI:** 10.1155/2015/174178

**Published:** 2015-03-02

**Authors:** Jamshed Iqbal, Mariya Al-Rashida, Serdar Durdagi, Vincenzo Alterio, Anna Di Fiore

**Affiliations:** ^1^Department of Pharmaceutical Sciences and Centre for Advanced Drug Research, COMSATS Institute of Information Technology, Abbottabad, Pakistan; ^2^Department of Chemistry, Forman Christian College (A Chartered University), Ferozepur Road, Lahore 54600, Pakistan; ^3^Division of Biophysics, Department of Basic Medical Sciences, School of Medicine, Bahçeşehir University, Turkey; ^4^Institute of Biostructure and Bioimaging (IBB), The Italian National Research Council, Via Mezzocannone 16, 80134 Naples, Italy

Carbonic anhydrases (CAs, EC 4.2.1.1) are metalloenzymes which are ubiquitous in nature and are found in a variety of organisms. In mammals at least 16 different isozymes of CAs have been found. CAs catalyze the reversible hydration of carbon dioxide to bicarbonate with the release of protons. Abnormal levels and/or activities of these enzymes have been associated with many disorders such as glaucoma, obesity, gastric ulcers, acid-base imbalances, cancer, and epilepsy. Carbonic anhydrases, therefore, have emerged as a valuable drug target for treatment or prevention of these disorders. Many clinically established drugs are CA inhibitors, and it is highly anticipated that many more will eventually find their way into the market. Much development has been made in this field; however, in order to find isozyme selective inhibitors with increased CA inhibition activity, it is necessary for new classes of compounds to be screened. This special issue has been dedicated to showcasing recent developments made in the field of CA inhibitors.

In “Carborane-Based Carbonic Anhydrase Inhibitors: Insight into CA II/CA IX Specificity from a High-Resolution Crystal Structure, Modeling, and Quantum Chemical in Calculations,” P. Mader et al. report crystal structure of CA II in complex with 1-methylenesulfamide-1,2-dicarba-closo-dodecaborane at 1.0 Å resolution. Using computational chemistry techniques, they then modelled the same carborane-based inhibitor inside the active site of cancer related isozyme CA IX. This virtual model may provide helpful insights into the structure based design of other (more efficient and possibly selective) carborane-based CA IX inhibitors.

In “Sulfa Drugs as Inhibitors of Carbonic Anhydrase: New Targets for the Old in Drugs,” M. al-Rashida et al. have identified N-substituted sulfonamide containing drugs, the sulfa drugs, and their chlorotriazine derivatives as inhibitors of CA II. The trichlorotriazine derivatives of sulfa drugs are invariably more active inhibitors than their parent drugs. This study provides a rationale for investigating other derivatives of sulfa drugs able to act as selective inhibitors against various CA isozymes.

In “Saccharin Sulfonamides as Inhibitors of Carbonic Anhydrases I, II, VII, XII, and in XIII,” V. Morkūnaitė et al. have designed and synthesized a series of sulfonamide containing saccharin derivatives and investigated their CA inhibition activity against CA I, CA II, CA VII, CA XII, and CA XIII. Saccharin itself contains a secondary sulfonamide group and weakly binds to CAs. Introduction of another free sulfonamide group greatly increases the CA inhibition activity of saccharin derivatives. Many isozyme selective inhibitors were identified with binding affinities in nanomolar range.

In “Hydrophobic Substituents of the Phenylmethylsulfamide Moiety Can Be Used for the Development of New Selective Carbonic Anhydrase in Inhibitors,” G. De Simone et al. have reported the synthesis of a family of structurally related compounds containing a sulfamide moiety together with an inhibition study of these compounds for the CA isoforms I, II, IX, and XII. The X-ray structure of the cytosolic dominant isoform hCA II in complex with the best inhibitor of the series is also reported, providing insights into sulfamide binding mechanism to CAs. These results confirm that such zinc-binding group, if opportunely derivatized, can be usefully exploited for obtaining new potent and selective CA inhibitors.

In “Natural Product Polyamines That Inhibit Human Carbonic in Anhydrases,” R. A. Davis et al. have identified a series of naturally occurring polyamines, based on either a spermine or spermidine core, as inhibitors of CAs. Some of these compounds were found to be submicromolar inhibitors of cancer related isozyme CA IX. Interestingly, these naturally occurring compounds do not contain the typical zinc bind functional groups, which make up a large majority of CA inhibitors known. This paves way for exciting new opportunities to design and investigate CA inhibitors with alternate mechanism of inhibition that may or may not involve zinc binding.

In “Synthesis and In Vitro Inhibition Effect of New Pyrido[2,3-d]pyrimidine Derivatives on Erythrocyte Carbonic Anhydrase I and in II,” H. Kuday et al. have synthesized a series of indolylchalcones and pyrido[2,3-d]pyrimidine derivatives containing indole ring. All compounds were found to be able to inhibit CA I and CA II. These compounds represent an interesting class of nonsulfonamide containing CA inhibitors that need to be explored further to elucidate their mechanism of inhibition and to exploit structural features for the development of more effective and possibly selective CA inhibitors.

In “Binding of Carbonic Anhydrase IX to 45S rDNA Genes Is Prevented by Exportin-1 in Hypoxic in Cells,” E. Sasso et al. have provided evidence for regulated binding of CA IX to nucleolar 45S rDNA genes in human cells. In their efforts to reveal novel mechanisms in cell and cancer biology, the authors have described for the first time a function for CA IX and XPO1 (one of its major interactors) in nucleoli, highlighting a XPO1-based decoy mechanism. In particular, in hypoxic conditions the occurrence of CA IX/XPO1 complexes was related to decreased transcription of 45S rDNA genes. Such findings are helpful to unravel the complex hypoxic cancer cell biology and its inevitable link with CA IX.

In “Probing the Surface of Human Carbonic Anhydrase for Clues towards the Design of Isoform Specific in Inhibitors,” M. A. Pinard et al. have adopted a clever approach in their quest for design of isozyme selective CA inhibitors. In most of the alpha-CA isozymes the active site residues are highly conserved, presenting a particular challenge for the design of isozyme selective CA inhibitors. However, some variation in amino acid residues occurs towards the exit of the active site. A comparison of conserved and nonconserved regions of CA catalytic site of various CA isozymes provides a template by virtue of which these subtle differences can be exploited for the design of isozyme selective CA inhibitors.

*Jamshed Iqbal* *Mariya Al-Rashida* *Serdar Durdagi* *Vincenzo Alterio* *Anna Di Fiore*

